# Decolonizing health in Canada: A Manitoba first nation perspective

**DOI:** 10.1186/s12939-021-01539-7

**Published:** 2021-09-15

**Authors:** Rachel Eni, Wanda Phillips-Beck, Grace Kyoon Achan, Josée G. Lavoie, Kathi Avery Kinew, Alan Katz

**Affiliations:** 1 Community Health Sciences, College of Medicine, University of Manitoba, Independent Researcher, Victoria, BC V9C 0M1 Canada; 2 Department of Community Health Sciences, Max Rady College of Medicine, Rady Faculty of Health Sciences, First Nation Health and Social Secretariat Manitoba and the University of Manitoba, Winnipeg, MB R3B 2B3 Canada; 3 Education Indigenous Institute of Health and Healing, University of Manitoba, Winnipeg, MB R3E 3P4 Canada; 4 Department Community Health Sciences, Max Rady College of Medicine, Rady Faculty of Health Sciences, University of Manitoba, Winnipeg, MB R3E 3P5 Canada; 5 First Nation Health and Social Secretariat Manitoba, Winnipeg, MB R3B 2B3 Canada; 6 Department of Family Medicine and Community Health Sciences, Max Rady College of Medicine, Rady Faculty of Health Sciences, University of Manitoba, Winnipeg, MB R3B 2B3 Canada

**Keywords:** First Nation health, Healthcare, Decolonization, Self-determination, Population health, Indigenous health, Primary healthcare, Grounded-theory, Traditional medicine

## Abstract

**Introduction & Background:**

Global persistence of health inequities for Indigenous peoples is evident in ongoing discrepancies in health and standards of living. International literature suggests the key to transformation lies in Indigenous efforts to control Indigenous health and healthcare. Previous authors have focused upon participation, structural transformation, and culturally appropriate healthcare recognized as a political right as fundamental tenets of Indigenous control. Contextualizing Indigenous health and wellness falls within a growing discussion on decolonization – a resituating of expertise that privileges Indigenous voice and interests.

**Methods:**

The study is a qualitative, grounded theory analysis, which is a constructivist approach to social research allowing for generation of theory in praxis, through interactions and conversations between researchers and participants. One hundred eighty-three interviews with additional focus groups were held between 2013-15 in eight Manitoba First Nation communities representing different models of health delivery, geographies, accessibilities, and Indigenous language groups. Community research assistants and respected Elders participated in data collection, analysis and interpretation. Line-by-line coding and constant comparative method led to the discovery of converging themes.

**Findings:**

Ultimately four main themes arose: 1) First Nation control of healthcare; 2) traditional medicine and healing activities; 3) full and meaningful community participation; and 4) cleaning up impacts of colonization. Joint analyses and interpretation of findings revealed substantial evidence that communities were looking profoundly into problems of improperly delivered services and health inequities. Issues were consistent with those highlighted by international commissions on reconciliation, health, Indigenous rights and liberties. To those documents, these findings add ground upon which to build the transformative agenda.

**Results & Discussion:**

Communities discussed the need for creation of protocols, constitution and laws to ensure growth of a decolonizing agenda. Inclusive to the concept are holistic, preventative, traditional health perspectives, and Indigenous languages. Colonization impacts were of critical concern and in need of undoing. Sharing of social and political efforts is seen as pivotal to change and includes all members of communities.

## Introduction & Background

Global persistence of health inequities for Indigenous peoples is evident in higher rates of illness and disease, food insecurity, living standards and mental health [1, 3, 4, 8, 13, 14, 16, 19, 33, 34]. International literature on health inequities suggests the key to successful outcomes is held within communities who have taken control over their health, health services and systems [12, 36]. Indigenous participation, engagement in health programming, policy development [37], structural transformation [38], and culturally appropriate healthcare, recognized as a political right [35] are specific tenets within those discourses of control.

Without control of healthcare there is a lack of clarity regarding unique perspectives, interests, histories, and contexts that Indigenous people bring to their healthcare experiences and the ways by which they inform optimal care provision for themselves and their families [7, 25, 32, 39, 40]. Also apparent is a growing focus on the problems of ongoing indigenous-specific racism, discrimination, the history of institutionalized healthcare and a lack of understanding of the unique life experiences of Indigenous peoples [32, 41].

Contextualizing Indigenous health and wellness falls within a growing broad discussion on decolonization of health and healthcare. In Canada and internationally, research and policy has focused on transferring control of healthcare to Indigenous governance [28, 29]. In Australia, Sherwood and Edwards recommended a necessary transformational shift in order to improve Indigenous health. Decolonization of healthcare systems, these authors emphasized, is necessary for this shift to occur. Decolonization includes resituating expertise such that Indigenous peoples become experts of their own health experiences, voicing and acting upon health initiatives. Furthermore, a systemic shift away from the ongoing dominant linear approach to health is needed [28]. Current knowledge from Indigenous communities highlights views on health and disease that are more inclusive and holistic, acknowledging the full spectrum of influences affecting wellness across the life course, aligning with traditional perspectives, e.g., access to and involvement in the production of healthy foods, physical activity, spiritual expression, and community empowerment [31] and among multiple levels of human existence, i.e., social, political and economic [11].

Our focus on decolonization stemmed from community discussions on underlying issues of healthcare provision and transformation efforts in on-reserve communities with effectively developed community-based primary healthcare models. First Nation active involvement and leadership in the research program shed light on elements of healthcare provision that constitute First Nation governance. Within effectively developed, community-based healthcare models was ample consideration to community-informed direction and interests, social determinants of health, holistic programming, traditional medicine, and jurisdictional bridging. By their very participation and involvement, solutions to complex and persistent issues seemed straightforwardly solvable.

This paper focuses on research of the Innovation Supporting Transformation in Community-Based Research Project (iPHIT) to learn from First Nations that have developed effective community-based primary healthcare models. It emphasizes data collected from interviews and focus groups pertaining to decolonization as fundamental to transformation, and ultimately to effective healthcare provision. Within decolonization, community representatives spoke on issues of self-governance, traditional medicine, holistic human and ecological wellbeing, full community participation and inter-governmental, inter-population collaboration. The beauty and richness of the research emanates from its methodology – from its reconsideration of expertise regarding the requirements of primary healthcare that is away from providers and institutions to the users of care.

### Defining decolonization in context

Decolonization addresses the multiple facets of disconnect between healthcare and Indigenous health outcomes, and the root of perpetual inequity itself. It is a process of reclamation of political, cultural, economic and social self-determination, including the re-development of positive individual, familial, community and nation level identities. Decolonization draws on colonial legacies, drawing on the knowledges and practices of pre-colonial, “traditional” times [17, 20]. These efforts require active involvement of Indigenous as well as non-Indigenous peoples (Mundel & Chapman). Decolonization has a revolutionary potential that requires the dismantling of colonialism as the dominant model upon which Canadian society [42], and healthcare provision, more specifically, operate.

## Methodology

### Background

This article is one of a program of research entitled ‘Innovation in Community-Based Primary Health Care (CBPHC) Supporting Transformation in the Health of First Nation and Rural/Remote Communities in Manitoba, Canada’ (iPHIT). The study was a 5-year research collaboration involving researchers from the University of Manitoba, 8 Manitoba First Nation communities, and the First Nations Health and Social Secretariat of Manitoba (FNHSSM), also recognized by its traditional name *Nanaadawewigamig,* which means “A Healing Place” in the Anishinaabe language. The Assembly of Manitoba Chiefs, Chiefs-in-Assembly, a political organization representing 63 Manitoba First Nations established the FNHSSM in 2013. The goal of FNHSSM is to create a unified health system with First Nations across the province through research, policy analysis, and advocacy. FNHSSM is also home to the Manitoba First Nations Health Information Research Governance Committee (MFN HIRGC), an ethical review body for all research involving First Nation peoples in Manitoba. MFN HIRGC reviews and approves research proposals to ensure that all research involving First Nation peoples meets 4 stipulated guiding principles: 1) free, prior, and informed consent at individual and collective levels; 2) First Nations OCAP principles, establishing First Nation ownership, control, access, and possession of data; 3) respect for First Nation ethical standards; and 4) benefit of the research to First Nations. These principles represent the essence of First Nation self-determination and governance in Manitoba [24].

The iPHIT program was one of 12 national teams funded via the Canadian Institutes of Health Research (CIHR) under the Community-Based Primary Health Care Initiative. Multiple challenges regarding provision of healthcare in First Nation communities, with a serious requirement for transformation in community-based primary healthcare was previously noted [18]. Contributing to the challenges for healthcare provision in the communities are small size of the communities, geographical isolation, and multiple jurisdictional operation of healthcare services and funding agencies: federal, provincial, regional health authorities, private for-profit organizations, and self-governing First Nation communities [24]. Further compounding delivery of healthcare is the multijurisdictional system for funding and delivery of community-based primary healthcare services, especially when the jurisdictions have not clearly defined each of their specific responsibilities, nor have they attended to colonization or its impacts [18, 24]. A growing evidence-base reveals that those First Nation communities who exercise control of their own healthcare appear to better meet the health needs of their community members. To further investigate this phenomenon, the iPHIT program focused on the experiences and strengths of community-based primary healthcare in First Nation communities, focusing on communities who have already begun their own healthcare transformation processes, utilizing traditional values within community-based and population health promotion lenses. Further information on studies of the iPHIT program is published [15, 24, 41, 43–46].

### Partnership, establishment of goals and direction

The iPHIT program, developed in partnership between FNHSSM and the University of Manitoba, built upon engagement and the capacities of the First Nations with the following overall goals in mind: 1) to describe community-based primary healthcare provision in First Nation communities by focusing on their strengths, key factors and innovations in healthcare to maintain wellness of community members; 2) to explore First Nation perspectives regarding why mainstream approaches to health may be failing; 3) to compare governance models, community engagement and delivery processes in and between the communities; and 4) to build collaborative relationships with communities and decision-makers in support of community-based primary healthcare innovation implementation to improve overall wellness of First Nations (see also [24]). These questions guided the qualitative study from which multiple articles have already and are in the process of being published [15, 24, 41, 43, 44, 46]. For the iPHIT team, the process of collaborative and respectful engagement was pivotal for implementation of a decolonizing methodology.

### Analytic strategy

We followed the general theoretical assumptions of grounded theory (GT) as described by Charmaz and others in her footsteps [5, 6]. GT is a constructivist approach to social research that allows for generation of theory in praxis, through interactions and conversations between researchers and research participants [30]. The theoretical approach advocates use of sensitizing concepts, which Charmaz defines as background notions that inform the research problem and provide the lenses through which we see, organize and understand experience. Sensitizing concepts become rooted within our conceptualizations of what is and how things ought to be in the world through ideological constructions and relative interpretations of reality. Though they may deepen perceptions, “they provide starting points for building analysis, not ending points for evading it.” In doing the work of GT, we use sensitizing concepts only as points for departure from which to study the data (2003: 259). The principles of GT provide a conceptual grounding while, at the same time, remain open to emergent themes [26].

The sensitizing concepts can be used further, to develop social constructs that are useful to studies in other social settings [2]. The constructs were derived from the perspectives of the research participants, from their language and expression, and that ‘sensitized’ the researchers to possible lines of inquiry [10]. Social constructs within Indigenous and decolonized methodologies influenced the starting point for our inquiry and analysis. Though we committed to adhering to GT methodology, that is to cast judgement and preconceived notions aside, we can only do so to a certain conscious extent beyond which we become blinded by our biases. All of the researchers went into this study perceiving that colonization continues to influence inequities in health and that racism exists and affects Indigenous peoples at every level of healthcare.

Linking back, the reviewed literature and previous studies by the research team indicated that the basic theoretical argument was that decolonization was an essential step to health equity for Indigenous peoples that included concepts such as taking back control of health, reclaiming and reviving health, and community-based participation. We assumed these concepts contained the theoretical notions that would set up the context for the overall research program. Although the goal for the research was to induce theory from the data, we are all to some extent influenced by our immersion in the politics of Indigenous health.

The sensitizing concepts emerged from the First Nations’ desire to transform primary healthcare in their communities by taking back control over their healthcare in order to improve accessibility, relevance and overall health and wellness. In initial discussions about development of the iPHIT research program, community participants addressed concepts pertaining to a necessary transformation, which included self-determination and autonomy, consensus and active participation in healthcare design and delivery, and well as addressing key issues, such as racism and colonial imposition. These sensitizing concepts informed the interview and focus group question guides.

### First nation collection of first nation data

Discussions about the research program began with a focus group meeting involving community leaders, health representatives and community members. As well, a regional conference was held, which allowed each participating community to share developments of their primary healthcare models. Each community identified a local research assistant (LRA) to serve as lead researcher for their own community. LRA’s attended a research assistant training program and were ultimately responsible for sharing information about the study, recruitment, planning interviews and focus groups, data collection, co- analysis and interpretation, validation, and dissemination of findings and results.

### Participant recruitment

Eight of Manitoba’s 63 First Nation communities were invited to participate in the study. The communities were chosen purposefully, representing different models of health service delivery and 4 of the 5 First Nation languages in the province: Ojibway, Cree, Dene, and Dakota. The communities are geographically dispersed throughout Manitoba, with four each in the north and south. Community sizes range from small (a few hundred residents) to large (a few thousand residents). Two communities are isolated, accessible only via fly-in or winter ice roads. Two are semi-isolated, accessible by road but are far away from city centres. Four communities are rural with all-season road access. The four northern communities have nursing stations and the southern communities have health centres. All receive federal funding for healthcare. All came into the study with unique and innovative perspectives on community-based primary healthcare.

Within each community, LRAs utilized a snowball approach to recruitment of participants for interviews and focus groups. LRAs approached individuals they thought would have interest and insights into healthcare system experiences. One person spoke to another in the community about their involvement in the study and soon approximately 10 from the smaller communities, 20-30 from medium sized, and 50 from the larger communities were interviewed. Focus group discussions began as LRAs and researchers began to look into, discuss and analyze the data. As such, sensitizing concepts began to emerge and to shape conversations about the data. The focus groups helped to keep a momentum and conversation in communities about the research.

### Interviews and focus groups

A total 183 interviews were held between 2013 and 2014. Community focus group discussions were held in 2014 to 2015. An interview guide developed in discussions between LRAs and the researchers, following the initial regional meetings, was used. The interview guide included open-ended questions with probes to encourage in-depth responses. Interviews and focus groups were in confidential spaces following individual participant comfort, such as at their homes, in the community health centres, or outside. Interviews lasted 90-120 minutes in length. Focus groups were 2-2.5 hours each. In each community, LRAs guided the discussions. Data collected from the interviews helped shape the focus group discussions. Eight to 15 individuals participated in each of the focus groups.

Participants ranged in ages, including young and older adults. There were equal numbers of men and women participants. Health directors and other healthcare providers from the communities were also participants. Although socioeconomic demographics were not recorded, participants represented lower to middle income brackets, a spectrum of educational level, from no secondary to college or university and graduate education. Elders brought a historical and traditional wisdom with them to the discussions.

Interview questions were organized into several categories regarding health needs, interests, service availability, accessibility, and more fundamental aspects of health and delivery of healthcare programming for First Nations, i.e., philosophy, history of colonization and self-government. Questions were included pertaining to personal and familial experiences in the different levels of healthcare, from community to tertiary or emergency care, and on biomedical as well as traditional knowledge and resources. Interconnections between jurisdictions, ecologies and areas of expertise were also brought into the discussions. Participants were seen as experts of their health experiences and were invited to share their wisdom in order to add substance to a transformative agenda, which it was hoped, would encourage vast improvement in self-governance, healthcare, and ultimately health of First Nations.

### Analysis

All discussions within each community were audio-recorded and transcribed verbatim. In total, 400 pages of transcribed data were collected. Data was collected until saturation was reached. Meaning that through conversations and reviews of the data between LRAs and researchers, we had come to realize that information is becoming repetitive and no new information is being said. Once the data from all of the communities was collected, files were imported into Nvivo 10 software. . Excel was also used to assist in organizing observations from the data and to record the emergent themes. Transcripts were rechecked for accuracy against the audio-recordings.

Analyses included line-by-line coding and constant comparative method by which newly collected data is compared to former existing data in order to derive new codes, themes, and conceptual focal points. We looked for converging themes by community and then question by question. As we looked across communities, ideas and themes started to emerge. Patterns and interrelationships began to emerge. Codes were grouped into the following 4 themes: (1) First Nation control of healthcare; (2) traditional medicine and healing activities; (3) full and meaningful community participation; and (4 cleaning up impacts of colonization.

To perform a GT analysis in collective, we set up levels of analysis that began with community level validation or rechecking of the data, having the LRAs check in with participants to ensure accuracy of interview and focus group transcripts, and then later, checking with the participants to verify interpretation of the data. LRAs and researchers compared data sets, allowing common themes to emerge. Weekly meetings were held until 2017, led by one or two of the researchers (GKA and RE). Ongoing discussions allowed for a growing intimacy with the data, bringing themes to life and revealing a cohesive story of decolonization of health in the region. In 2018, a regional conference was held – bringing together and cohesively validating the research.

Trustworthiness of qualitative data is a standard concept used in qualitative research that is in contrast to conventional, positivistic criteria for external and internal reliability, validity and objectivity [2]. At community and regional levels, we employed trustworthiness techniques including: member checking, negative case analysis and thick description. Ultimately, a plausible a coherent explanation of issues pertaining to the topic emerged. Validation was achieved by presenting the results back to participants and the communities in community and regional workshops.

### Ethical overview

Ethics approvals were sought and obtained from the University of Manitoba Research Ethics Board and from the Health Information Research Governance Committee (HIRGC), which is supported by the FNHSSM and is responsible for ethics reviews of all research proposals involving First Nations. Consent to participate was communicated through Band Council Resolutions made by each participating community, after meeting by project team members. The research adhered to the First Nation principles of ownership, control, access and possession of data (OCAP), which gave the communities decision-making authority regarding the details of information collection, utilization, and management [9].

## Findings

Joint analyses and interpretation of the findings between researchers and First Nation people revealed substantial evidence that the communities were looking profoundly into the problems of poorly delivered healthcare services and health inequities. Issues brought to the fore were consistent with those highlighted by national and international commissions regarding reconciliation, health and wellbeing, Indigenous rights and liberties. What the community members generated through the interviews could be described aptly as substance to conceptual images conceived in central discursive circles. Essentially, we were all on the same page regarding it being high time colonialism and all it entails be brought to its head. However, the work that the communities were doing was providing an actual ground upon which to build a decolonizing, and therefore, transformative agenda. A culmination of work that can be described as dissecting decolonizing into pieces of not only coming out of a colonized mindset but re-entering into and revival of traditions, that included values, aspirations, and ways of doing health work that were outside of what has for over a century been shaped by colonial and, inside of that, biomedical power and control.

### First nation control of healthcare

The first theme involves a realization that someone must be controlling what gets done in healthcare, including what gets prioritized, attended to, and ignored. Community members believed it was necessary to inquire about one’s roles and responsibilities in order to create change. If one is not actively involved in decision-making about health matters that involve self and family, it is important to ask how this has come to be so or, perhaps, where that authority has gone lost. Interviewees across the communities agreed that control over such matters was taken from them by the Canadian governments. Consequently, they agreed in the necessity of resuming that control.

First Nation control and ownership would provide better access and would have the biggest effect on community health (A027).

Government has just conditioned us too much as Aboriginal people... They want to control us, and they want to stop where we are (wanting to move forward). Now we need to start empowering our people with knowledge and power and give back to take ownership of their communities, their health (GFG004-4).

What we need is less interference from government. Like right now, we are simply agents of the federal government. We administer their programs. We need access to resources which we can control and be accountable for, but control as we see fit (C008).

We need to have more awareness and more participation from individuals themselves, patients themselves. I believe they need to be more accountable for their own health (C016).

Self-governance begins with the family. The healthy part is to have vision by the community. We want the community to be healthy and it has to start from the self and then family and then community – just having the opportunity to make decisions and for communities to create their own guides for wellness (D005).

We need to get over what was imposed on us, those laws. One way… is creating our own constitution, creating our own laws. The Province of Manitoba and the government of Canada have to ensure, the economic capabilities of First Nations are supported, we need room to grow. The best way of doing that is by giving First Nation people opportunity to govern (D014).

First Nation models of health and wellbeing were holistic, including multiple domains of human development – physical, emotional, mental/cognitive and spiritual, as opposed to biomedicine’s focus on the anatomical/physiological body. Further, healthcare included attention to how individuals live within their multiple ecologies, including what they eat, how they hunt, gather, and prepare their foods. Paying attention to prevention and health promotion strategies would go a further way than medical surgeries in resolving chronic disease epidemics.

Prevention! We are so focused on intervention right now, it’s tough to turn to prevention, but we’re taking steps in the right direction and if we can just front load our prevention models, the better it will be because that means we’re doing everything we can to provide tools and skills to emerge to address those health issues before they become life threatening (D014).

Decolonizing the mind and the way the mind is interpreted, to participant interviewees, meant having thoughts and behaviours understood and respected from within a perspective that acknowledges the values and meaningful existence, what it means to be a First Nation person.

We need more psychiatry, but psychiatry based in our society. They come in and they don’t understand our society, the way the society is (A007).

To decolonize requires an emphasis by communities to develop local expertise, working within communities, and according to traditional methods of health provision and understandings about the human condition.

We would have our own band members as physicians, which we might be close to having in the next couple of years. A lot of people in the health centre are our own band members, which is fantastic. A lot of First Nations communities don’t have that yet (D014).

Quality healthcare is to have a good nursing station with all of the programs that are needed, NNADAP, mental health, nurses, medications, having all of those services here, all complete… A lot of lives can be saved just by keeping people here instead of sending them away so much (C011).

To train people better, work together with the community in our way and I think you’d have something substantial. I think now the people lack capacity (C011)

Permanence is essential, permanent… not temporary nurses… (C011).

Decolonizing spaces as well as work patterns were aspects of taking back control of health, as the following comments suggest.

The (community health representatives) need to get out of the offices, go door-to-door and see about the people in their homes, home visits, taking healthcare to the people and embracing health as a community (A007).

We’re all scattered. We’re not in one building or space as health workers. The nursing station is over there, social services is over here, and the other health workers are in a different area too. Sometimes no one knows what’s going on (A005).

### Traditional medicine

Communities saw colonization as a system of beliefs and practices imposed upon them, at the same time devaluing, ridiculing and forbidding traditions. Biomedicine halted development of First Nation medicines and healing philosophies. Now, the participant interviewees said, it was time to bring these knowledges back to the forefront of healthcare.

We would have traditional medicines brought back at the nursing stations for people to use. Our people are losing their limbs from diabetes and this isn’t supposed to happen. I have that medicine for that so that won’t happen to them. They won’t have to lose their limbs (C012).

The main thing, the fear of the traditions has to be erased (A005).

Too many people now don’t believe in our culture. They don’t even believe in traditional values anymore. There’s been a conflict, I guess, between white society and our own culture. There’s just a lot of people have been brainwashed into thinking that culture is bad. You don’t want to see a medicine man because generally, people think it’s bad to go to one. And when somebody does go, they think they’re practicing black magic or something like that. So, people tend to stay away from something like that (A007).

We have to re-educate our people on our own traditional medicines. We have a lot of learn and to differentiate, which herbs to use for spirituality and for physical health and so on. Educating to the purpose of the benefits of traditional uses of medicines is important. A lot of these chemically produced medicines have a lot of side effects. We need to get the people that have knowledge of these traditional medicines to explain the benefits of the traditional medicine, he herbs, and so on, bringing down the disillusionment that the community may have of using such traditional medicines (C005).

Revival of First Nation traditional knowledge would involve collaboration between the communities.

We can have a guide, a guide that would be for the community or for all the nations. If we’re going to have our own guide, I guess to have some understanding, there’s only certain things that are growing or could be grown in this community. In other communities there’s only things that can be grown there. So, a guide would be a good thing to begin with. Like, we’re got access to this, this is what we can grow and that is how you can use it, stuff like that. Because you have a lot of Manitoban communities, they do come pick sweet grass here, even from Saskatchewan, Alberta, Ontario, and British Columbia (the other provinces). They actually travel all that way to come and pick the medicines (D005).

Elders in each of the communities are thought to hold pertinent knowledge about traditional health.

It’s more than the medicine men or medicine people involved in spirituality or have knowledge of such medicines. It’s our Elders have a working knowledge of these medicines but they are not being utilized so we need to promote education, give them a place to share, in a working group, groups together that are interested in medicines and further our understanding, utilize what we have and study (C005).

Reconnecting after colonization was seen as pivotal to reclaiming health and healthcare. Traditional medicine and healing activities presupposed human-ecological interconnection. Importantly, what was interrupted with by the colonial regime was still accessible to the people, it may be hidden, in need of rediscovery, but discoverable, ready to be revitalized, nonetheless.

My dad was torn apart from his land and traditions. When I came back here, I was filled by that, all of the traditions and family that I missed out on. I never wanted to leave, the sense of community, the sense of the land and the people. When I do leave, even for a day, I get lonely, I get physically sick. We’re safe here and you feel it, you’re at home, on the land, something with the land, a bond, something (A007).

Our medicine people are coming back strong. The western doctors, they did so much harm than good (D012).

### Full and meaningful community participation

Participant interviewees highlighted the fundamental importance of full participation in community development matters. Everyone had a voice. Personal and unique experiences of each gender and age group were vital to the creation of the type of healthcare services that would attend to the needs of the people. This type of participation is inherent in a First Nation conception of self-determination.

It’s an integrated system we need that delivers services based on the health needs of the community (A027).

Most of our problems are about social issues. People are hurting, suicidal, heart-broken parents, these need to be understood and addressed, people need a place to just be heard and to be (A029).

We need to listen to the kids, our youth, because they are the ones that are going to take over this health centre one day. If these kids get healthier and more cultural that will be such a positive thing in the community (B005).

Open dialogue, I’m looking at this question from our oral tradition, which is more or less for me, open dialogue. Get the feedback from the people, the ones that are receiving the healthcare and what they think can be improved (C005).

People get stuck and it’s only one person making all the decisions, it’s got to be everybody, the whole community to say, “Let’s get on board, let’s help one another, support one another and make those changes as a community” (F006).

The vision should be created in consultation with community. So many times, the community is left out (F1).

With the older people that are out there now, I would want… the youth to do that… go and visit the Elders… Visit them, learn something from them while they’re still here… We need to get their input and learn. We need to get back some of that what the Elders had in how they grew up and how these youth have grown up that which are two different things all together! Learn from each other because some of these older people, even a lot of young people don’t know medical things… It is time consuming, but you can learn and teach in discussions (C010).

### Cleaning up impacts of colonization

Whether or not participant interviewees focused on a need for more biomedical intervention brought closer to the communities, or even, into communities, or for improved health and wellness via holistic, health promotion and preventative strategies, one thing was agreed upon – colonization has reeked havoc on individual, family and community life, so much so that all facets of life and living must be attended to in order to revive health. Healthcare in First Nation communities would have to delve into the SDH, issues of self-governance, and human-ecological interactions – across the life span. The multiple perspectives are evident in the following comments.

Our population has grown in the last few years. This place needs a hospital, should have had a hospital years ago (A002).

More resources instead of having to go out of the community for care, such as x-rays (A008).

We have different doctors coming and going but we need to have a permanent doctor stationed here (C003).

By far, not all of the research participants agreed that the antidote to the serious health inequities in Canadian society was more biomedicine. Most focused their discussions on the SDH, preventative measures, and on cultural traditions in order to create healthcare programming in line with First Nation perspectives on health.

There are lot of ailments in this community, major deficiencies regarding health and there is a disequilibrium of wealth. There is a lot of abject poverty and with poor health conditions. I don’t think access to healthcare is equitably distributed. Negative dynamics like drugs, alcohol, solvent abuse and dysfunction in peoples’ lives (C016).

When it comes to health, it’s not just the physical health of the person, it’s a whole lot of things that surrounds that person and preventing them from becoming healthier. We can focus on those things (D014).

We have to go back to the land. Going back to the land means going back to culture. Chopping wood, making a fire… we have to bring that stuff back (A007).

Reconnecting with the land is all a part of health and the governments can support us on that (A0101-2).

## Results and discussion

Participants spoke to decolonizing health in their communities through four interconnecting themes. First Nation control of healthcare, implied full and engaged responsibility in attending to community needs and interests. Topics included the creation of protocols, constitution and laws that ensure room to grow and economic capabilities. Control of healthcare would encompass holistic, preventative approaches to healthcare, a decolonized mind and decolonized spaces, reviving richness of the traditional languages and deep understanding of the nourishing potential of the local expertise.

Next, participants saw colonization as an external imposition, squashing cultural traditions, manifesting fear through historical manipulations at multiple levels. They saw need for a re-education and revival, inclusive of every individual in the communities. They spoke of the need to establish safety in reconnecting to the land, its resources and to one another.

Full participation was discussed as a concept unlike consensus in Western discourses, rather, as it involves engagement in health transformation by all people, representing a diversity of perceptions, experiences and interests by all genders and ages. Full participation is a foundational requirement of self-determination.

Finally, moving forward was seen to necessitate a cleaning up of debris left behind by colonization. Participants spoke at length about the havoc colonization has reeked upon them personally and upon multiple generations within their communities. They spoke to the need to delve into SDH, self-governance, and human-ecological interactions that were affected across the lifespan. Inequitable distribution of resources, imbalances of power, lack of access to societal supports were the culprits to abject poverty, drug and alcohol addictions, and poor health across the spectrum.

The participatory research study gathered voices of First Nation community members on the topic of transforming primary healthcare practices, ultimately towards improving the health of First Nations. The stories, comments, and ideas culminated in an action-oriented response to decolonization. Across the communities, people knew what they needed in order to live a healthy life – to participate in the making of that healthy life. Contrasting the knowledge gained through data analysis raised a very important question – have we complicated health so much so that we have destroyed the simple logic of how to live a healthy life? It seems power and governance issues have had such detrimental impacts on humanity as to cause discernable differences based on race, class – the creation of boundaries between human beings. Participant interviewees shared community struggles in taking back their wisdoms about the land and healing properties of substances that come from it. Colonization shaped people to doubt themselves, to feel shame about the very things meant to keep them well in the world, in their governance, and in their interactions between others and the natural environment.

Wisdom, after colonization, was a privilege only of those who earned degrees from colonial institutions. In this way, Elders stopped sharing what they knew, and medical men and women almost went out of existence. Community members at large were not asked what they thought nor invited to participate in health development activities. Instead, they learned to consult with experts outside of themselves and their cultures. All of these losses were discussed. All shaped the SDH, the poverty, the serious addictions to drugs and alcohol that we see today.

In discussions on decolonization, participant interviewees talked of the need to encourage self-determination within the communities. They emphasized the value of full community engagement with respect to inclusion of different interpretations of and experiences in the world. They highlighted the creation of shared vision for health, a notion that is consistent with previously published research [21, 22]. Self-determination is a capacity realized in common by members of a distinct political community, working together within shared political institutions to determine laws and policies that will share their individual and collective futures [21]. Engagement, for the participant interviewees, was essential for self-determination. Community empowerment developed out of a shared respect for the engaged work of community members – a kind of vitalizing medicine that develops from within.

The focus on full community participation in planning, knowledge sharing and decision-making is also in keeping with previous published research. For example, Smylie et al. [27] reported that local investments in all aspects of healthcare including planning, community perceptions of the programs as intrinsic, otherwise stated as having claimed a sense of ownership through high levels of engagement, are linked to positive health results.

Decolonizing health means clearing and taking back in a sensible and instinctual way, power. It involves a sharing of power – the power to know, based on being in the world and a power to do, according to one’s learned and sensual interactions with physical environments. The participant interviewees shared deep thoughts about reviving lost, hidden, and denigrated knowledges. Importance of cultural continuity, revival, and the relationship between preservation, health, and self-determination was studied previously [47, 23].

## Limitations

The data presented represents the perspectives of participants in the 8 communities studied. It does not presume to speak for the other 63 Manitoba First Nation communities, nor elsewhere nationally and internationally. However, to the extent that all Indigenous peoples have experiences colonization, the study is relevant across the globe.

## Conclusion

The paper focused on the work of community members from 8 Manitoba First Nation communities to decolonize health in Manitoba, Canada. Decolonization as a concept is consistent with the work of national and international commissions discussed in the background section of this paper. The participant interviewees, guides to our overall research program, informed on the nuts and bolts of decolonizing – the how to undo colonialism and its detrimental impacts.

From a decolonizing lens, this research adds an important dimension to advancing the knowledge-base as it raises the voices and iterates the perspectives and opinions of First Nation people – healthcare workers, directors, community leaders, Elders and members at large – on a grave and timely topic. Decolonization, as the participants of this research describe it, essentially involves all members of the communities, their wisdoms, and life experiences. Future research will only strengthen our understanding about how to move past colonization to greater inclusion, development of traditional medicines, and of being in and interacting with the natural environment. Ultimately, the data gathered over the course of this research will offer meaningful and transformative strategies for improvement of primary healthcare in First Nation communities, by empowered First Nation communities.

## Data Availability

All data used for this analysis are protected under privacy policies of the data stewards of FNHSSM, HIRG and within the terms of the institutional review board approval for this study, and are not publicly available.
